# A recurrent missense variant in the E3 ubiquitin ligase substrate recognition subunit FEM1B causes a rare syndromic neurodevelopmental disorder

**DOI:** 10.1016/j.gim.2024.101119

**Published:** 2024-03-07

**Authors:** François Lecoquierre, A Mattijs Punt, Frédéric Ebstein, Ilse Wallaard, Rob Verhagen, Maja Studencka-Turski, Yannis Duffourd, Sébastien Moutton, Frédédic Tran Mau-Them, Christophe Philippe, John Dean, Alice S. Brooks, Marjon A. van Slegtenhorst, Julie A. Jurgens, Brenda J. Barry, Wai-Man Chan, Eleina M. England, Mayra Martinez Ojeda, Elizabeth C. Engle, Caroline D. Robson, Michelle Morrow, A. Micheil Innes, Ryan Lamont, Matthea Sanderson, Elke Krüger, Christel Thauvin, Ben Distel, Laurence Faivre, Ype Elgersma, Antonio Vitobello

**Affiliations:** 1 Univ Rouen Normandie, Inserm U1245 and CHU Rouen, Department of Genetics and reference center for developmental disorders, F-76000 Rouen, France.; 2 UMR1231 GAD, Inserm, Université Bourgogne-Franche Comté, Dijon, France.; 3 Department of Clinical Genetics, Erasmus MC, 3015 GD Rotterdam, The Netherlands.; 4 ENCORE Expertise Center for Neurodevelopmental Disorders, Erasmus MC, 3015 GD Rotterdam, The Netherlands.; 5 Institut für Medizinische Biochemie und Molekularbiologie (IMBM), Universitätsmedizin Greifswald, 17475 Greifswald, Germany.; 6 Nantes Université, INSERM, CNRS, l’institut du thorax, 44007 Nantes Cedex 1, France.; 7 Unité Fonctionnelle Innovation en Diagnostic Génomique des Maladies Rares, Fédération Hospitalo-Universitaire-TRANSLAD, CHU Dijon Bourgogne, Dijon, France.; 8 Laboratoire de Génétique, CHR Metz-Thionville, Hôpital Mercy, Metz, France.; 9 Department of Medical Genetics, NHS Grampian, Aberdeen, UK.; 10 F.M. Kirby Neurobiology Center, Boston Children’s Hospital, Boston, MA, USA.; 11 Department of Neurology, Boston Children’s Hospital, Boston, MA, USA.; 12 Department of Neurology, Harvard Medical School, Boston, MA, USA.; 13 Broad Institute of MIT and Harvard, Cambridge, MA, USA.; 14 Howard Hughes Medical Institute, Chevy Chase, MD, USA.; 15 Center for Mendelian Genomics, Program in Medical and Population Genetics, Broad Institute of MIT and Harvard, Cambridge, MA, USA.; 16 Analytic and Translational Genetics Unit, Massachusetts General Hospital, Boston, MA, USA.; 17 Division of Genetics and Genomics, Boston Children’s Hospital, Boston, MA, USA.; 18 Department of Ophthalmology, Boston Children’s Hospital and Harvard Medical School, Boston, MA, USA.; 19 Division of Neuroradiology, Department of Radiology, Boston Children’s Hospital, Boston, MA, USA.; 20 Department of Radiology, Harvard Medical School, Boston, MA, USA.; 21 GeneDx, Gaithersburg, Maryland, USA.; 22 Alberta Children’s Hospital Research Institute for Child and Maternal Health and Department of Medical Genetics, Cumming School of Medicine, University of Calgary, Calgary, AB T2N 4N1, Canada.; 23 Department of Medical Genetics, University of Alberta, Edmonton, Alberta, Canada.; 24 Centre de référence maladies rares « déficiences intellectuelles de causes rares », Centre de Génétique, FHU-TRANSLAD, CHU Dijon Bourgogne, Dijon, France.; 25 Centre de Référence maladies rares « Anomalies du Développement et syndromes malformatifs », Centre de Génétique, FHU-TRANSLAD, CHU Dijon Bourgogne, Dijon, France.; 26 These authors contributed equally.

**Keywords:** FEM1B, neurodevelopmental disorder, neurogenesis, ubiquitination, reductive stress, p.(Arg126Gln)

## Abstract

**Purpose:**

FEM1B acts as a substrate recognition subunit for ubiquitin ligase complexes belonging to the CRL2 E3 family. Several biological functions have been proposed for FEM1B, including a structurally resolved function as a sensor for redox cell status by controlling mitochondrial activity, but its implication in human disease remains elusive.

**Methods:**

To understand the involvement of FEM1B in human disease, we made use of Matchmaker exchange platforms to identify individuals with *de novo* variants in *FEM1B* and performed their clinical evaluation. We performed functional validation using primary neuronal cultures and *in-utero* electroporation assays, as well as experiments on patient’s cells.

**Results:**

Five individuals with a recurrent *de novo* missense variant in *FEM1B* were identified: NM_015322.5:c.377G>A NP_056137.1:p.(Arg126Gln) (FEM1B^R126Q^). Affected individuals shared a severe neurodevelopmental disorder with behavioral phenotypes and a variable set of malformations, including brain anomalies, clubfeet, skeletal abnormalities, and facial dysmorphism. Overexpression of the the FEM1B^R126Q^ variant but not FEM1B wild-type protein, during mouse brain development, resulted in delayed neuronal migration of the target cells. In addition, the individuals’ cells exhibited signs of oxidative stress and induction of type I interferon signaling.

**Conclusion:**

Overall, our data indicate that p.(Arg126Gln) induces aberrant FEM1B activation resulting in a gain-of-function mechanism associated with a severe syndromic developmental disorder in humans.

## Introduction

Ubiquitination is a post-translational modification that involves the covalent attachment of one or more ubiquitin molecules onto a target protein. This acts as a signal for protein degradation through the ubiquitin-proteasome system (UPS). The final ubiquitin-ligation step is performed by E3 ubiquitin-ligases which function either as single proteins or multi-protein complexes. One family of ubiquitin ligases are the Cullin-RING E3 ubiquitin ligases (CRLs), which are multi-protein complexes consisting of a scaffold protein (cullin) that recruits a RING-box protein (encoded by *RBX1* (MIM 603814) or *RNF7*, also known as RBX2 (MIM 603863)) and an adaptor protein that can interact with several substrate recognition proteins, ensuring the specificity of the reaction^1^. The CULLIN 2-based E3s (CRL2s) are involved in many biological processes including cancer, germline differentiation, and viral defense^1,2^. Several recognition subunits of CRL2s have been described, including von-Hippel Lindau tumor suppressor (*VHL*, MIM 608537) and SWI/SNF proteins *ARID1A/ARID1B* (MIM 603024 and 614556), in which heterozygous variants lead to familial cancer syndromes or developmental disorders, respectively^3^. Similarly, *FEM1B* (Fem1 homolog B, MIM 613539) is a CRL2 substrate recognition subunit with a VHL-box for assembly with the other components of the E3 complex: Elongin C (*ELOC*, MIM 600788) and Cullin 2 (*CUL2*, MIM 603135)^4^. FEM1B is a ubiquitous protein, localizing mainly in the cytoplasm, but studies based on cellular fractionation have also suggested a role in the nucleus^5^.

Several functions of *FEM1B* include roles in apoptosis^6,7^, glucose homeostasis^8^, secondary sexual development^9^, replication stress pathways^5^, and more recently reductive stress pathways^10,11^. Although few FEM1B-substrate interactions have been resolved at the molecular level, FEM1B has been shown to interact with distinct types of amino-acid sequences, termed degrons. *CDK5R1* (MIM 603460) is an example of FEM1B target that has such a degron on their carboxy-terminal (C-degron proteins)^12^. Binding to the CRL2-FEM1B complex initiates the C-degron pathway leading to the breakdown of these proteins^13,14^. Recently, *FEM1B* was shown to be involved in the regulation of the redox state of cells by mediating the ubiquitination and C-degron-independent breakdown of the reductive stress sensor *FNIP1* (MIM 610594)^10^. This reaction was shown to be under the control of proteins encoded by the BEX gene cluster^11^.Despite the various identified functions of *FEM1B,* the association of the gene with human disease is less well defined. In this study we present the clinical and molecular findings of five patients harboring a recurrent *FEM1B* missense variant. We show a disturbed redox state in patient’s cells and demonstrate the toxic effect of this variant on neuromigration in developing mice. We further discuss the molecular mechanism in light of recent fundamental studies on *FEM1B* biology.

## Materials and Methods

The study was performed within the framework of the GAD (“Génétique des Anomalies du Développement”) collection and approved by the appropriate institutional review board of Dijon University Hospital (DC2011–1332).

### Individuals and sequencing

Individuals with an unexplained developmental disorder and a *de novo* variant in *FEM1B* were identified via data sharing through GeneMatcher^15^, Matchmaker exchange, and using the denovo-db database^16^ as previously described^17^. Clinical and molecular information was collected by the referent clinical geneticists. Ancestry information (supplementary information) was assigned by clinicians. Written informed consent for study participation was obtained by qualified individuals. Data were collected in accordance with ethical guidelines of participant inclusion centers and accordance with the Declaration of Helsinki.

### Mice

We obtained pups to set up primary neuronal cultures by crossing congenic FvB/NHsD male and female mice. In utero electroporations were performed on pregnant FvB/NHsD females that were crossed with C57BL/6J males. All experiments performed in this study were approved by the Dutch Central committee for animal experiments (CCD; approval AVD101002017893) and by the local review board, and in accordance with the European Commission Council Directive 2010/63/EU and compliant with the ARRIVE guidelines.

### Primary neuronal cultures and established cell lines

The procedure for preparing primary hippocampal and cortical neuronal cultures has been detailed elsewhere and will only briefly be addressed below^18^. Hippocampal and cortical tissue from embryonic brains (E16.5) was separately collected in 10 mL neurobasal medium (Gibco, 21103–049) Following a 20-minute incubation at 37 °C in prewarmed trypsin/EDTA solution (Sigma, T3924–500 mL) the material was dissociated in 1.5 mL NB medium (supplemented with 2% B27, 1% penicillin/streptomycin and 1% GlutaMax (Gibco, 350500–38)) using a 5-mL pipette. The dissociated neurons were plated on poly-D-lysine (Sigma, p0899) 25 mg/ml)-coated glass coverslips (15 mm) at a density of 1 × 10^6^ cells per well, in a 12-wells plate.

HEK 293T cells were cultured in DMEM GlutaMAX (Thermo Scientific, 10569010) supplemented with 10% (v/v) fetal calf serum (FCS) and 1% penicillin and streptomycin. All cell lines used in this study were grown at 37 °C in a 5% CO_2_ humidified incubator.

### Plasmids and site-directed mutagenesis

We amplified the entire *FEM1B* (NM_015322.5) coding sequence from human cDNA using the following primers: *Fw* 5’- GGCGCGCCTATGGAGGGCCTGGCTGGCTATG-3’ and *Rv* 5’- GCGGCCGCTTAATGAAATCCAACAAACTCTTCAAGAGTTC -3’. The *Fw* primer contained a 5’ AscI site, and the *Rv* primer a 5’ NotI site, to facilitate cloning into our dual expression vector. This expression construct allows for the simultaneous expression of *FEM1B*, under the control of the CAGG promotor, and *tdTomato* from the PGK promotor. Similar expression constructs have been previously described and used^19–22^. In this study we have taken along a control construct without a FEM1B insert but still expressing tdTomato, henceforth referred to as Empty Vector. The FEM1B p.(Arg126Gln) missense variant was generated with the Quickchange Lightning Site Directed Mutagenesis Kit (Agilent, 210513) using the two following primers: *Fw* 5’- CAAAGCATGCTGCCTGCAGGGGGGTTGAA -3’ and *Rv* 5’- TTCAACCCCCCTGCAGGCAGCATGCTTTG -3’.

### Transfection of HEK cells

One day prior to the transfection, 2 × 10^5^ HEK 293T cells were seeded into each well of a 12-well plate. The following day, we transfected each well with 1.5 μg plasmid DNA using polyethylenimine (PEI). To this end, plasmid DNA was taken up in 100 μl serum-free DMEM, to which an equal amount of serum-free DMEM was added, containing PEI in a ratio DNA (μg) : PEI (μl) of 1 : 3. The entire mix was incubated at room temperature for 15’ before being added to each well in a dropwise manner. The cells were placed back at 37 °C for four hours, whereafter the transfection medium was replaced with fresh culturing medium. The cells were harvested, lysed, and FEM1B protein stability was analyzed 48 hours after transfection by Western blotting.

### Western blot for protein stability

Transfected HEK 293T cells were lysed in lysis buffer (250 mM Sucrose, 20 mM HEPES [pH 7.2], 1 mM MgCl_2_, 10 U/mL Benzonase, and protease inhibitor (complete Protease Inhibitor Cocktail (Roche 11836145001). Total protein concentration was determined using the BCA Protein Assay Kit (Life Technologies Europe, 23225), whereafter a total of 20 μg of protein per sample was loaded and run on an 18-well 4–15% Tris-Glycine gel (Bio-Rad, 567–1084). The separated proteins were transferred to a 0.2 μm nitrocellulose membrane (Bio-Rad, 170–4159), which was subsequently blocked for 30 minutes in TBST (10 mM Tris-HCl [pH 8.0], 150 mM NaCl, 0.1% Tween-20 (Sigma, P1379)) containing 5% (w/v) skim-milk powder (Sigma, 70166). Primary antibodies (human FEM1B, HPA041920, 1:1000, Sigma), (tdTomato, #600401379, 1:2000; Rockland),(human GAPDH, 2118S, 1:1000, Cell Signaling) were dissolved in TBST and incubated on the membranes at 4 °C. The following morning, the membrane was briefly rinsed and incubated a secondary Li-Cor antibody (goat anti-rabbit 680 CW, 1:15,000, LI-COR Biosciences, 926–68073). After an hour at room temperature, the membrane was thoroughly rinsed (3 wash steps with TBST and 3 wash steps with TBS) and analyzed using the Li-Cor Biosciences infrared fluorescence scanner. The ImageStudioLite software (RRID:SCR_013715) was used to quantify protein bands. FEM1B protein levels were normalized against tdTomato expression and the levels of FEM1B^WT^ protein were set at a 100%.

### In-utero electroporation experiments

We performed the in-utero electroporation experiments according to previously described protocol^22^. An overview of the method is presented on www.functionalgenomics.nl. Succinctly, we anesthetized pregnant FvB/NHsD mothers bearing pups of E14.5 gestational stage and surgically exposed the uterus. Leaving the uterus intact, we used a glass pipette controlled by the Picospritzer^®^ III device to inject 1–2 μl plasmid DNA (1.5–3 μg/μl) in the lateral ventricle of the embryo. Tweezer-type electrodes, connected to a pulse generator (ECM 830, BTX Harvard Apparatus), were then used to provide five electrical square pulses of 45 V of 50 ms each and 150 ms interpulse interval. The electrodes were placed such that the progenitor cells in the somatosensory cortex were targeted. We injected Empty Vector, FEM1B^WT,^ and FEM1B^R126Q^ expression vectors to assess the effect of mutant FEM1B overexpression on neuronal migration. The pups were sacrificed at P1 after which the brains were subjected to histochemical processing.

### In-utero electroporation histochemistry

We performed transcardial perfusion with 4% paraformaldehyde (PFA) on mice that were injected (i.p.) with an overdose of Nembutal. After extraction and post-fixation in 4% PFA, we embedded the brains in gelatin. Using cryoprotective sucrose buffer (30% sucrose in 0.1 M phosphate buffer (PB)), we froze the embedded brains on dry ice and made sections using a freezing microtome (40–50 μm thick). Sections were washed in PBS and blocked in PBS containing 10% normal horse serum (NHS) and 0.5 % Triton X-100 for 1 hour at room temperature. We then incubated the slices with a primary RFP antibody (#600401379, 1:2000; Rockland) dissolved in PBS containing 2% NHS, 0.5% Triton X-100 at 4°C for 48–72 h. Slices were washed three times with PBS after which the secondary antibody was added (Cy3 donkey-anti-rabbit, 1:400; Jackson ImmunoResearch) diluted in PBS containing 2% NHS, 0.5% Triton-X 100. Finally, the slices were counterstained with 4′,6-diamidino-2-phenylindole solution (1:10,000; Invitrogen) before being mounted on glass slides using Mowiol. Overview images of the coronal sections were acquired by tile scan imaging using an LSM700 confocal microscope (Zeiss) with a ×10 objective.

The obtained images were rotated such that the number of cells in different cortical layers could reliably be counted using ImageJ (Analyze Particles option). We divided the cortical areas, starting from the pia and ending at the ventricle, in 10 equally sized bins, in which the percentage of tdTomato-positive cells were counted. The confocal images used for this analysis came from two to three non-consecutive sections from at least three animals per plasmid. This procedure has been described in more detail elsewhere^22–24^.

### Neuronal transfection and immunocytochemistry

After being in culture for 7 days (DIV7) we transfected our primary neurons using Lipofectamine 2000 (Invitrogen, 11668–019). Prior transfection, neurons were transferred to a new 12-well plate, containing a transfection mix (neurobasal medium, supplemented with glutamine (500 μM)), and the conditioned medium was stored at 37°C, 5% CO2 for later use. A mixture containing complexed DNA-Lipofectamine (1.8 μg DNA per well) was added dropwise to the neurons, followed by a 1-hour incubation at 37 °C, 5% CO_2_. Afterward, neurons were transferred to the conditioned medium and placed back at 37 °C, 5% CO_2_.

We fixed neurons 5 days after transfection using 4% PFA/4% sucrose. The coverslips were rinsed with PBS and before mounting the coverslips on microscope glass slides, we incubated them for 15’ in 4′,6-diamidino-2-phenylindole solution (DAPI, 1:10000, Invitrogen). Our slides were mounted using Mowiol-DABCO (Sigma) mounting medium and confocal images were acquired using an LSM700 confocal microscope (Zeiss).

We analyzed neuronal morphology of at least 10 confocal images (20× objective, 0.5 zoom, 1024 × 1024 pixels) of different transfected neurons. tdTomato expression in transfected neurons enabled their identification. The complexity of branching was analyzed using Scholl analysis and individual dendrites and branches were traced using the NeuronJ plugin in ImageJ. For our Scholl analysis, we counted the number of neurite intersections between the traced neuron and a series of concentric circumferences at an interval of 10 μm from each other, with the center on the soma.

### Statistics

All statistical analyses were performed using Graphpad Prism (v7.0 for Macintosh, GraphPad Software Inc., RRID:SCR_002798). Normality tests were performed on the data of individual experiments, and where this was not met, non-parametric tests statistics have been used. All data are represented as mean ± SEM, unless specified otherwise.

### Individual T cells

Peripheral blood mononuclear cells (PBMCs) used in this study were isolated from whole-blood from individuals as well as related healthy family members (father and/or mother of the proband) or unrelated individuals by PBMC spin medium gradient centrifugation (pluriSelect). To expand T cells, collected PBMCs were cultivated in U-bottom 96-well plates together with irradiated feeder cells in RPMI 1640 supplemented with 8% human AB serum (both purchased from PAN-Biotech GmbH) in the presence of 150 U/ml IL-2 (Miltenyi Biotec) and 1 μg/μl L-PHA (Sigma) following the procedure of Fonteneau et al. (ref^25^). After a 3-week culture, resting T cells were washed and frozen as dry pellets for further use.

### SDS-PAGE and western blot analysis

T Cell pellets were subjected to protein extraction by resuspending them in equal amounts of standard RIPA buffer (50 mM Tris pH 7.5, 150 mM NaCl, 2 mM EDTA, 1 mM N-ethylmaleimide, 10 μM MG-132, 1% NP40, 0.1% SDS) and soluble protein lysates were quantified using a standard BCA assay (Thermo Fisher). Ten micrograms of total proteins were denatured with 6% SDS and subsequently treated with 2,4-dinitrophenylhydrazine (DNPH) to modify protein carbonyl groups using the OxyBlot Protein Oxidation Detection Kit from Merck Millipore. Reaction was stopped by adding a 0.375-fold volume of neutralization solution (2 M Tris, 30% glycerol) and 1 mM DTT and proteins were separated by 10% SDS-PAGE prior to western-blotting using an anti-DNP primary antibody. Carbonylated proteins were then visualized using anti-goat secondary antibodies (1/5,000) and enhanced chemiluminescence detection kit (ECL) (Bio-Rad).

### Gene expression analysis by Nanostring

Total RNA was isolated from snap frozen T cell pellets derived from Subject 1 and healthy donors using the innuPREP RNA Mini Kit from Analytic Jena AG following the manufacturer’s recommendations. One hundred nanograms of total RNA was subsequently used for hybridization to Nanostring nCounter^®^ Human AutoImmune Profiling Panel covering 750 predefined immunological relevant genes. Gene expression was normalized to a combination of housekeeping genes and heatmaps of differentially expressed genes were generated using nSolver 4.0.

### Gene expression analysis by RT-qPCR

Total RNA was extracted from T cells using the kit from Analytic Jena AG following the manufacturer’s recommendations prior to reverse transcription using the M-MLV reverse transcriptase (Promega). To determine the mRNA expression levels of each IFN-stimulated gene (ISG), quantitative PCR (qPCR) was performed using the Premix Ex Taq^™^ (probe qPCR) obtained from TaKaRa. The qPCR analysis was conducted in duplicate, and FAM-tagged TaqMan^™^ Gene Expression Assays purchased from Thermo Fisher Scientific were used for ISG quantification, following the manufacturer’s instructions. The TaqMan^™^ probes employed in this study included IFI27, IFI44L, IFIT1, ISG15, RSAD2, IFI44, and MX1. To assess the relative expression of the target genes, the cycle threshold (Ct) values were converted to relative expression values using the relative quantification (RQ) method, specifically the 2-ΔΔCt calculation. The expression of the target genes was normalized to the Ct values of the housekeeping gene GAPDH, which served as the control.

## Results

### Identification of a *FEM1B* recurrent missense variant in patient with NDDs

The first genetic evidence for a developmental disorder associated to FEM1B was identified within a previous work on recurrent *de novo* variants^17^ using the denovo-db database^16^. In this study we identified in our cohort of almost 5000 exomes a female individual with a *de novo* missense variant in *FEM1B* NC_000015.9:g.68582073G>A NM_015322.5:c.377G>A NP_056137.1:p.(Arg126Gln) previously identified by the Deciphering Developmental Disorders (DDD) project^26^. This variant is absent from population databases including gnomAD V2.1 (~280,000 alleles) and the UK biobank (276,334 alleles) and is predicted deleterious by several missense predictors such as polyphen2^27^, MutationTaster^28^, SIFT^29^, and CADD^30^. Analysis of the missense tolerance ratio (MTR) metrics^31^ showed that p.(Arg126) was located within the most constrained region of *FEM1B* (tolerance score dn/ds 0.04, Figure S1). This residue also showed high phylogenetic conservation. Subsequently, data sharing through GeneMatcher^15^ enabled the identification of additional unrelated individuals with the same *de novo* CpG transition in *FEM1B*, leading to a total of five individuals (four females, one male). All affected individuals had been seen in genetic counseling in the context of a sporadic developmental disorder, and data analysis did not identify additional strong candidate genes or variants. Of note, individual 4 underwent both research-based and clinical exome sequencing (Supplementary information), both of which independently highlighted *FEM1B* as a candidate gene. The variant in individual 5 showed an allelic ratio of 26% (73/283), suggesting post-zygotic mosaicism (Figure S2).

### Human genomics and animal model data support a non-haploinsufficient mechanism

The FEM1B protein is composed of 627 residues encoded by two exons. Despite its small size, LoF alleles are observed in gnomAD v2.1, resulting in a loss-of-function observed/expected upper bound fraction (LOEUF) metric of 0.52. This indicates that there is no significant depletion of LoF variants in the general population, making haploinsufficiency of FEM1B unlikely. In line with this assumption, previous studies have shown that heterozygous and homozygous depletion of FEM1B in mice did not affect viability or produce developmental/malformation phenotypes^8,9^. Moreover, recurrence of the same *FEM1B* missense variant in all five affected individuals suggested a non-haploinsufficient mechanism^32^. Recurrent missense variants are often associated with gain-of-function or dominant-negative mechanisms, as seen in *FGFR3* (MIM 134934), *PACS1* (MIM 607492), *PACS2* (MIM 610423), *AKT1* (MIM 164730), *SMAD4* (MIM 600993), *PPP2R5D* (MIM 601646), and *MTSS2* (MIM 616951), among others^33–39^. Altogether, the identification of the exact same ultra-rare missense variant occurring *de novo* in five unrelated individuals with developmental disorder was a very strong signal for a novel disease with a suspected non-haploinsufficient mechanism based on human genomics data and animal models.

### Clinical phenotype of FEM1B related NDD

**Figure 1:**
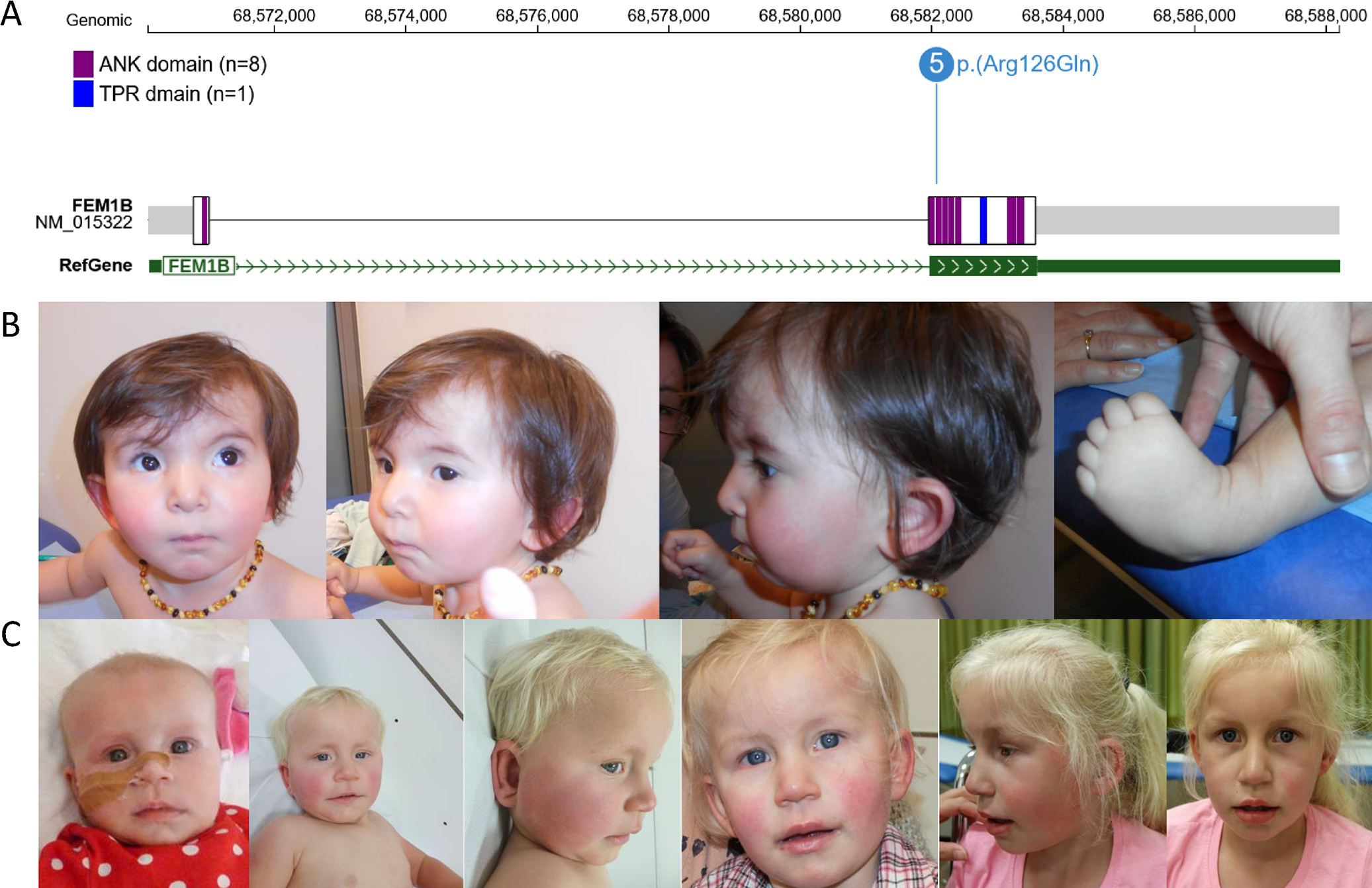
Clinical and molecular aspects of FEM1B p.(Arg126Gln) associated disorder (A) Genomic (GRCh37) and protein contexts showing FEM1B p.(Arg126Gln) recurrent variant. Made on ProteinPaint using domain information from Uniprot (web resources). Of note, individual 5 harbors the variant in the mosaic state with an allelic ratio of 26%. (B) Morphologic features of Individual 1 at age 14 month. Note the presence of dysplastic ears, epicantus, flat nasal bridge and hypoplastic alae nasi. (C) Morphologic features of Individual 3. Note the mild hypertelorism, upslanted palpebral fissures, arched eyebrows, wide mouth, thick vermillion border, long grooved philtrum, reddish cheeks and mildly low-set ears. (D) Morphologic features of Individual 5. Note the deep set eyes, dysplastic cupped ears, broad nasal root and pointed chin.

The clinical data of the five individuals are summarized in Table 1 and Figure 1. Each individual was seen in a medical genetics consultation to assess a suspected genetic developmental disorder. Their age at last consultation ranged from 3.5 to 25 years. All individuals were born at term and had normal prenatal ultrasounds. Significant malformations were noted at birth in Individuals 1 and 3, who both presented with talipes equinovarus and congenital heart defects affecting the auricular or ventricular septum, which required surgery in Individual 3. Individual 1 was diagnosed with pyloric stenosis and underwent surgical treatment on day 15. All individuals presented with global developmental delay. The average age at which individuals sat unassisted was 13 months and walk unassisted was 3.8 years. Language acquisition was also delayed, and all five individuals developed mild to moderate intellectual disability. Behavioral abnormalities were observed in four individuals, with features of autism such as stereotypies, rituals, and overstimulation. Strikingly, these four individuals (1 to 4) exhibited significant auto-aggressivity, including self-biting and head banging.

Brain malformations were reported in three individuals (3 to 5), involving corpus callosum in three and cerebellum in two. Individual 3 was noted to have ponto-cerebellar and corpus callosal hypoplasia. In Individual 4, corpus callosum and cerebellar involvement were also observed, along with a periatrial gray matter heterotopia, arachnoid cyst, and mild dysmorphic enlargement of the ventricles. Additionally, Individual 4 exhibited spinal cord involvement in the form of a syrinx or cyst at the C6-C7 level. Individual 5 exhibited a thin corpus callosum and a mild cerebral atrophy. Scoliosis was diagnosed in three individuals and was severe enough to require surgery in two. Fused cervical vertebrae (C2 + C3) were observed in Individual 4. Individual 4 also had severe hip dysplasia that required extensive treatment which had a major functional impact. Hearing loss was noted in two individuals and refractive errors in three individuals. Individual 4 exhibited significant ophthalmological involvement, including severe myopia with posterior staphyloma and optic nerve atrophy. Additionally, individual 4 had limited adduction of the left eye caused by a 6th nerve palsy. Dermatological features were noted in the individuals, including thin or sparse hair in three individuals and significant Raynaud’s phenomenon in two individuals as recurrent symptoms. Precocious puberty was observed in two of the three older individuals, while the other two were too young to confirm, suggesting that it may be a prevalent symptom. Other significant medical histories included severe central sleep apnea, Hashimoto’s thyroiditis, and eosinophilic esophagitis, all of which were present in the oldest individual, Individual 4. Facial dysmorphic features were noted in all five individuals (Figure 1, Table 1). Recurrent dysmorphic features included plagiocephaly (2/5), a square or prominent forehead (2/5), epicanthal folds (2/5), deep set eyes (2/5), and a wide mouth (2/5). Morphological abnormalities of hands and feet were also reported in all individuals including slender fingers, clinodactylies, toes syndactylies, and camptodactylies. The growth parameters were mostly within normal range, except for the cranial perimeter of Individual 2 at age 6 which showed macrocrania, and the presence of an excessive weight gain in Individual 5 who developed severe obesity after early childhood (Table S2 and Figure S3). Comprehensive clinical details are available in Table S1 and individual case reports are present in the Supplementary information. Though the variant was mosaic in Individual 5, her phenotype was broadly comparable to the other individuals.

### FEM1B p.(Arg126Gln) overexpression affects neuronal migration and morphology

We then set out to assess the effect of p.(Arg126Gln) variant on neurodevelopment. According to human brain span atlas the gene is expressed throughout human brain development till adult and its expression is relative constant. According to the Allen mouse brain atlas expression is highest in neurons of the hippocampal formation, cortical subplate and cortex. This information and the hypothesis of a gain of function mechanism prompted us to use murine models of (i) *in utero* electroporation and (ii) primary hippocampal neurons in order to look for developmental and structural neuronal anomalies in mutated context.

We first generated an expression construct of wild type FEM1B (control) and FEM1B p.(Arg126Gln). Transfection of the FEM1B constructs into HEK293 cells revealed that the protein levels of the FEM1B^R126Q^ were comparable to that of wild type FEM1B, suggesting that the p.(Arg126Gln) variant does not affect protein stability (Figure S4).

**Figure 2:**
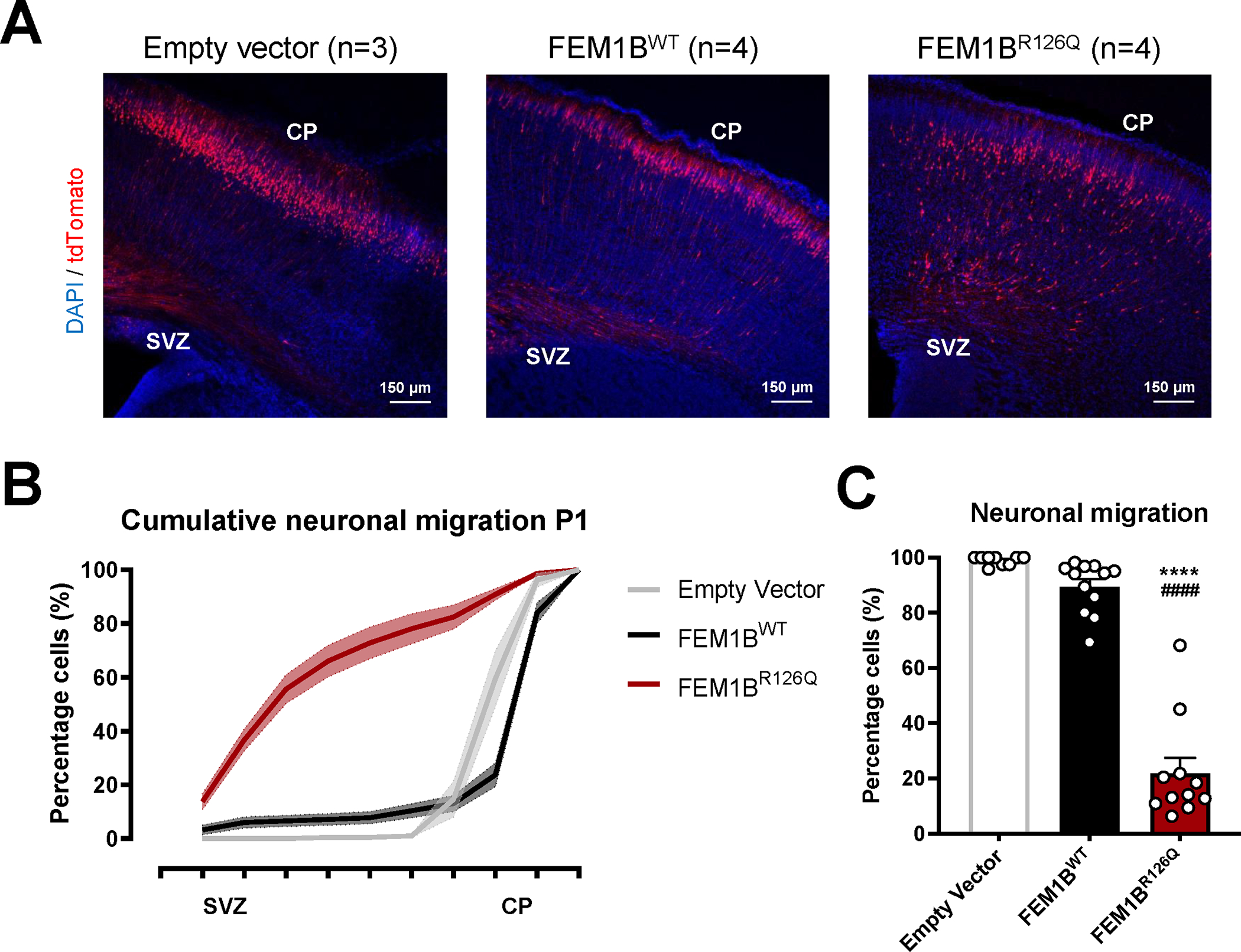
In utero electroporation of FEM1B^R126Q^ results in a neuronal migration deficit in P1 pups (A) Representative images from FEM1B in utero electroporation (IUE) at postnatal day 1 (P1). Neurons in the subventricular zone (SVZ) were electroporated at embryonic day E14.5 with an expression vector (Empty Vector, FEM1B^WT^ and FEM1B^R126Q^, all expressing tdTomato), and their migration towards the cortical plate (CP) was visualized at postnatal day 1(P1). (B) Cumulative distribution of transfected neurons between SVZ and CP, as counted in 10 bins between the SVZ and the rim of the cortex. (C) Percentage of cells that successfully migrated to the cortical plate (defined by the sum of last 4 bins of graph B). Averages were compared with a one-way ANOVA and Tukey’s *post hoc* test. *Compared with Empty Vector ^#^compared with FEM1B^WT^. **** p < 0.0001, ^####^ p < 0.0001. All data are presented as mean ± SEM.

Next, we used *in utero* electroporation to assess the impact of the FEM1B variant on neuronal development, maturation and migration. We have previously shown that this assay, which measures migration of the sparsely targeted neurons from the subventricular zone (SVZ) to the outer layers of the cortical plate (CP), is very sensitive to expressing mutated proteins that affect neuronal function^21,23,24^. The FEM1B constructs (wild type and FEM1B p.(Arg126Gln)), alongside with a control vector, were overexpressed in brains of E14.5 (embryonic day 14.5) mice by *in utero* electroporation, and neuronal migration was evaluated at P1 (postnatal day 1). Histological analysis showed that overexpression of p.(Arg126Gln) but not wild type FEM1B significantly delayed neuronal migration of the targeted cells as compared to the non-targeted cells (Figure 2).

**Figure 3:**
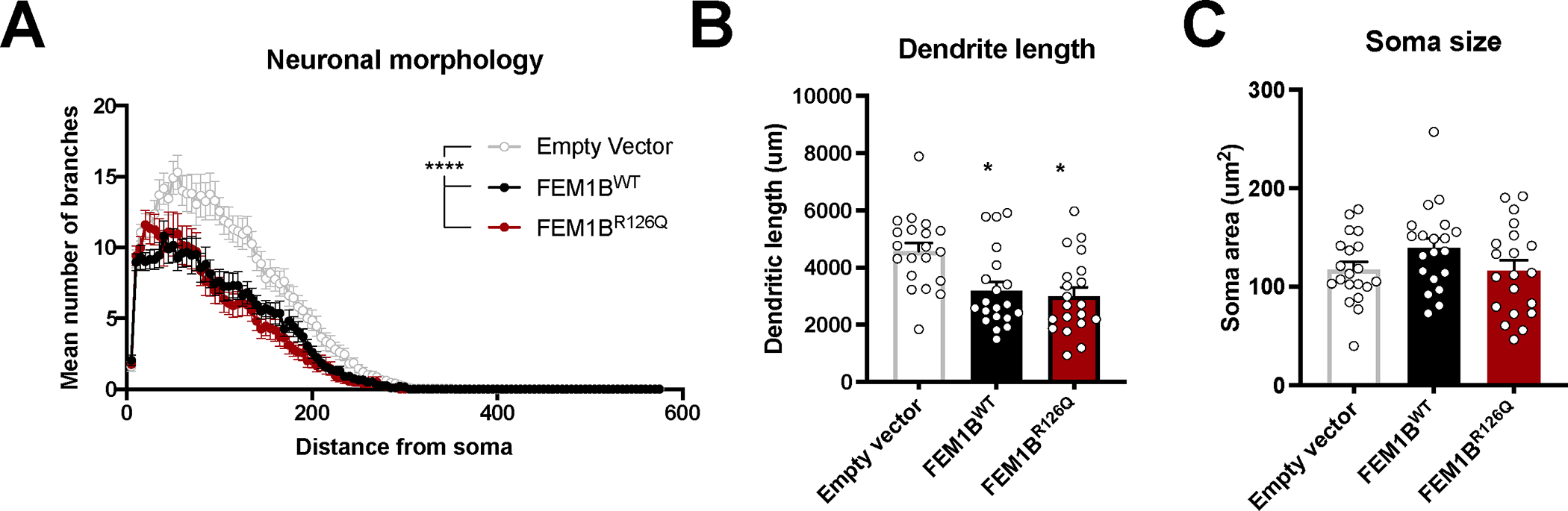
Overexpression of FEM1B^WT^or FEM1B^R126Q^ affects neuronal morphology. (A) Sholl analysis of neurons transfected with FEM1B^WT^ and FEM1B^R126Q^ variant. The degree of branching of each transfected neuron was measured by drawing circles of increasing size around the cell soma and counting the amount of intersecting dendritic branches. (B-C) The average dendritic length (B) and soma size (C) of neurons transfected with Empty Vector, FEM1B^WT^ or FEM1B^R126Q^. *compared to Empty Vector using one-way ANOVA and a Bonferroni *post hoc* test. *p < 0.05. All data are presented as mean ± SEM.

To test the effect of wild type and mutant FEM1B on neuronal maturation, we transfected primary mouse hippocampal neurons at DIV7 (Days In Vitro) with the FEM1B constructs using an empty vector as control and analyzed 3 days after transfection and analyzed dendritic length and soma size. These experiments showed no statistically significant differences between wild type and FEM1B^Arg126Gln^ groups. However, both wild type and mutant FEM1B led to a significant decrease in both dendritic length and arborization relative to the empty vector control (Figure 3), suggesting that increased FEM1B expression or activity is detrimental for proper neuronal development. Taken together with the cellular migration experiments, these results support the hypothesis that the FEM1B p.(Arg126Gln) variant acts through a gain-of-function mechanism impacting normal brain function.

### T cells derived from patients carrying the R126Q variant exhibit a cellular pattern of oxidative stress

**Figure 4:**
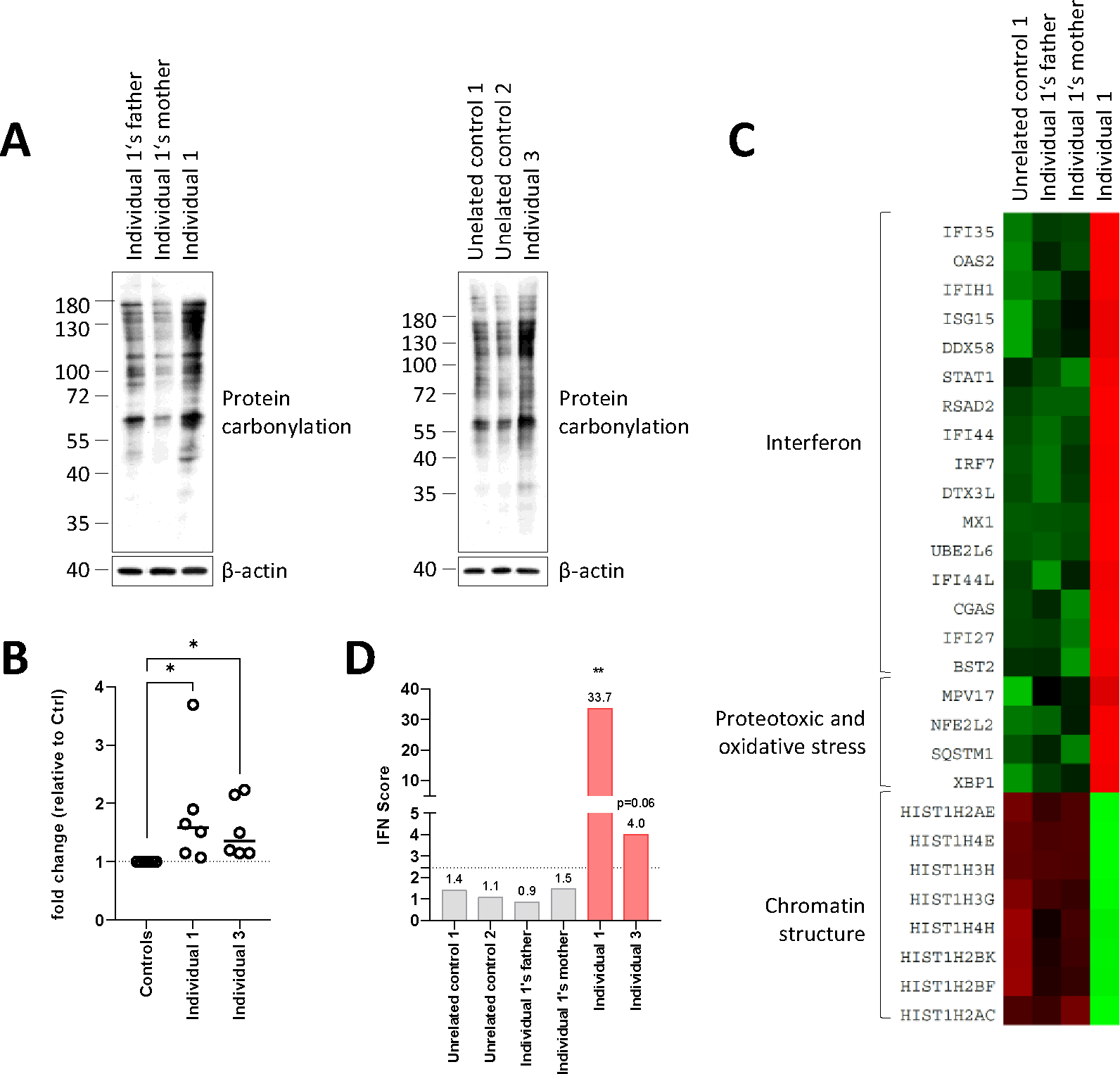
T cells from individuals with FEM1B p.(Arg126Gln)-associated disorder exhibit signs of oxidative stress. (A) Oxyblot experiment. T cells isolated from Individuals 1 and 3 were compared to related parental controls and unrelated controls, respectively, for their content in carbonylated proteins by SDS-PAGE/western-blotting using an antibody specific for DNP. Equal protein loading was ensured by probing the membrane with a monoclonal antibody directed against β-actin. Shown is one representative experiment out of three. (B) Densitometry analysis of the western blots depicted in A. Data are presented as fold changes to control samples whose densitometry measurements were set to 1 (gridline) after normalization with loading controls. Shown are median values from three independent experiments. Statistical significance was assessed by Wilcoxon signed-rank test (*P<0.05). (C) Transcriptomic profiling. Heat mapping of fold change of gene expression between T cells derived from Individual 1 and a control group consisting of T cells derived from the proband’s parent and from one unrelated healthy donor (adult male), as indicated. Upregulated and downregulated genes are represented in red and green, respectively. (D) IFN signature by RT-qPCR. IFN scores for controls and FEM1B Individuals 1 and 3 were calculated as the median of the relative quantification (RQ) of seven interferon-stimulated genes (ISGs) over a single calibrator control. Statistical analysis was performed using paired Student t test with ** indicating *P*<0.01 vs controls.

T cells offer a distinct advantage of being the only *in vitro* proliferating cells obtainable from blood specimens, affording us a substantial quantity of material for conducting crucial functional assays on primary cells. It is also noteworthy that a mounting body of evidence from numerous reports suggests a notable involvement of the immune system, specifically neuroinflammation, in the pathogenesis of NDD^40–46^. FEM1B has been shown to be involved in reductive stress, mitochondrial integrity and the preservation of the cellular redox balance^10,11^. To confirm the relationship between the *FEM1B* R126Q potential gain-of-function variant and oxidative stress, T cells from Individuals 1 and 3 were assessed for free carbonyls, whose formation is typically promoted under oxidative conditions^47^. Protein carbonylation was enhanced in both individuals relative to controls (Figure 4A). Quantification of three independent experiments confirmed that protein carbonylation was significantly higher in T cells carrying the *FEM1B* p.(Arg126Gln) variant than in their wild-type counterparts (Figure 4B).

To uncover gene signatures specific to FEM1B associated disorder, we next profiled 700 selected genes in T cells derived from Individual 1 and three heathy individuals (probands’ parents and one unrelated donor) using the NanoString nCounter^®^ technology, as previously described^48^. Heat mapping of relative gene expression in T cell samples derived from the affected individual revealed 20 upregulated genes, most of which belonged to the family of type I interferon (IFN)-stimulated genes (ISG), and 8 downregulated genes encoding core histones involved in nucleosome assembly (Figure 4C). Another smaller signature of genes upregulated in Individual 1 consisted of four transcripts (NFE2L2 (MIM 600492), SQSTM1 (MIM 601530), MPV17 (MIM 137960) and XBP1 (MIM 194355)) related to proteotoxic and oxidative stress. These observations are in line with previous work showing that the *FEM1B* p.(Arg126Gln) variant is associated with increased reactive radical oxygen species (ROS) production and oxygen consumption rate^11^. To further establish a potential link between *FEM1B* gain-of-function and the acquisition of type I IFN gene signature, we next examined the levels of ISG transcripts in T cells obtained from Individuals 1 and 3 by RT-qPCR. As shown in Figure S5, both patients exhibited increased transcription rates of the seven investigated ISG (i.e.; IFIT1, IFI27, IFI44, IFI44L, ISG15, MX1 and RSAD2) when compared to control samples. The notion that *FEM1B* gain-of-function triggers type I IFN responses was further substantiated by calculating the type I IFN scores using the methodology of Rice et al.^49^, which were found to be higher in both patients compared to controls (Figure 4D). Altogether, these data point to a substantial effect of *FEM1B* gain-of-function on cellular redox state which is accompanied by type I IFN and histone signatures.

## Discussion

In this study, we presented the phenotypical features of five individuals with a recurrent de novo *FEM1B* missense variant. We investigated the toxicity of this variant on the murine central nervous system, suggesting a gain-of-function mechanism. Finally, we demonstrated that patients exhibit signs of an impaired oxidation-reduction balance, offering insights into the pathological mechanism underlying this disease.

We demonstrated the deleterious effect of the p. Arg126Gln variant in the developing brain by making use of the *in utero* electroporation assay. This technique allowed us to target a small number of cells in the mouse brain, and to investigate the effect on neuronal migration upon either overexpressing the wild-type or the mutated FEM1B^R126Q^ protein as compared to the surrounding, untransfected (control), cells. Since the targeted cells have to compete with untransfected cells, this assay is very sensitive to changes that affect neuronal function, revealing more subtle abnormal migration behaviors than in the case of a germ-line variant. Our assay show that the p.(Arg126Gln) variant, acts through a GOF mechanism. This result was in line with the observation that, in our study, brain malformations were reported in three out of five cases (including heterotopia), indicating that the p.(Arg126Gln) variant affects structural brain formation at endogenous expression levels.

The role of FEM1B in the detection of reductive stress has been recently identified^10^ and studied at the molecular level through structural studies^11^. In this pathway, FEM1B ubiquitinates FNIP1 in response to reductive stress, resulting in the activation of mitochondria and the production of ROS^10^. This reaction was shown to be inhibited by the binding of proteins from the BEX family to FEM1B, which act as pseudo-substrate inhibitors for FEM1B’s activity^11^. The p.(Arg126Gln) variant was shown to alter the inhibition by BEX proteins, but did not affect the ability of FEM1B to activate mitochondrial metabolism^11^. These elements lead to a model in which mutant FEM1B is activated with low or absent reductive stress, resulting in aberrant oxidation compensation. Our study validates this gain of function mechanism by showing that T cells derived patients exhibit an increased pool of carbonylated proteins, reminiscent of oxidative stress (Figure 4B and 4C).

Remarkably, this was concomitant with the upregulation of NRF2 and SQSTM1/p62 (Figure 4A). NRF2 is a well-established transcription factor that evades proteasome-mediated degradation during oxidative stress by KEAP1 oxidation^50^. Given that NRF2 has the ability to activate both SQSTM1/p62^51^ and its own gene expression^52^, these findings suggest that FEM1B gain of function triggers activation of the NRF2/KEAP1 pathway and the subsequent induction of target genes associated with antioxidant and detoxification responses. These observations fully support recent reports demonstrating the coordinated regulation of the NRF2/KEAP1 and FNIP1/FEM1B pathways^53^. Interestingly, the elevated oxidant-damaged proteins detected in Individual 1 were accompanied by the induction of type I IFN-stimulated genes and repression of core histone genes (Figure 4A). Whether a cause-and-effect relationship between these two signatures exist remains unclear, but it is tempting to speculate that the loss of core histones may result in genomic instability and subsequent release of DNA fragments into the cytosol, which are then sensed as danger signals by the cGAS-STING pathway. Alternatively, given the adverse effects of oxidative stress on mitochondrial integrity^54,55^, the leakage of immunostimulant mitochondrial DNA and/or RNA into the cytoplasm is also conceivable. In this regard, the disruption of mitochondrial redox signaling and control by oxidative stress can also alter signaling pathways and is a known mechanism of congenital malformations^56^. It has notably been involved in the pathogenesis of heart and brain malformations and digestive atresia, reminiscent of the phenotypes we describe^56,57^.

The question arises whether other *FEM1B* variants could cause this disease. Because of the gain-of-function mechanism, only a small number of missense or related variants would be predicted to have this type of impact. We can conjecture that the lack of binding to BEX pseudo-substrate inhibitors without altered ubiquitination function is a very rare feature centered at codon Arg126. Of note, the p.(Arg126Ala) which was previously modeled^11^ and has the same effect as p.(Arg126Gln) is unlikely to happen in individuals because it cannot result from a single substitution of the CGG codon. In the eventuality of future variants of uncertain significance, Oxyblot analysis on PBMCs could be used as an easy functional test.

BEX proteins are encoded in a cluster of 5 genes located in the Xq22.2 region^58^, the heterozygous deletion of which have been involved in a developmental disorder termed early-onset neurological disease trait^59,60^. Seven females with a *de novo* deletion of at least one BEX gene have been described (individual 1 – 3 in ref^59^, BAB2595, BAB8120, BAB12522 and BAB2650 in ref^60^). Because the absence of BEX proteins could theoretically mimic the *FEM1B* p.(Arg126Gln) mechanism^11^, we compared the phenotype of both cohorts. The phenotype of Xq22.2 deletion was strikingly similar to the phenotype of *FEM1B* individuals. Like *FEM1B*, it consisted in a severe syndromic developmental disease with autism-like features and a variable set of malformations, including specific abnormalities like septal defect (BAB12522), talipes equinovarus (individual 2) and auto-mutilations and self-harming present in three individuals (BAB12522, individual 1, individual 3). Other less specific overlapping symptoms included hearing loss (BAB8120), thin hair (individual 3), hypothyroidism (BAB12522), esotropia (BAB2595, BAB2650, individual 2), and brain abnormalities and scoliosis (BAB8120). Overall the phenotypes of *FEM1B* p.(Arg126Gln) and Xq22.2 deletion of BEX proteins were mostly indistinguishable. A recent study suggested *TCEAL1* as likely involved in the phenotype of Xq22.2 deletion syndrome, but as most individuals with *TCEAL1* were hemizygous boys and because the phenotype did not recapitulate completely the Xq22.2 deletion phenotype, the pathogenic impact of BEX cluster deletion is still plausible. The identification of excess oxidation in these female individuals would be a strong argument further linking the two disorders.

In conclusion, we have identified a developmental disorder caused by a recurrent gain-of-function variant in the *FEM1B* gene. We showed that this variant caused an increase in oxidative metabolism and its overexpression affected neuronal migration in the developing mouse brain. While this disorder is likely ultra-rare due to the very limited mutational target, the understanding of the gain-of-function mechanism associated with *FEM1B* presents potential therapeutic opportunities. Molecules specifically targeting FEM1B have been developed^61^ which could be used to limit its activity.

## Supplementary Material

Supplementary Information and Figures

Supplementary Tables

## Figures and Tables

**Table 1:** Clinical and genetic findings in individuals with recurrent FEM1B variant p.(Arg126Gln)

		Count	Individual 1	Individual 2	Individual 3	Individual 4	Individual 5

	Sex		F	F	F	M	F

Lab and sequencing	*FEM1B* variant	−	NM_015322.4:c.377G>A p.(Arg126Gln)	NM_015322.4:c.377G>A p.(Arg126Gln)	NM_015322.4:c.377G>A p.(Arg126Gln)	NM_015322.4:c.377G>A p.(Arg126Gln)	NM_015322.4:c.377G>A p.(Arg126Gln)
	Transmission	−	de novo	de novo	de novo	de novo	de novo, mosaic in proband

Heart	Congenital heart defect	3/5	+	−	+	−	−

Abdomen	Pyloric stenosis	1/5	+	−	−	−	−
	Inguinal hernia	2/5	−	−	+	+	−
	Anteriorly placed anus	1/5	−	−	−	−	+

Central nervous system	Brain malformations	3/5	−	N/A	+	+	+

Musculo-skeletal	Scoliosis / vertrbral malformations	3/5	−	−	+	+	+
	Abnormal feet position	4/5	+	+	+	−	+
	Finger / toes abnormalities	5/5	+	+	+	+	+
Neuro-developement	Hypotonia	3/5	+	−	−	+	+
	Global developmental delay	5/5	+	+	+	+	+
	Sitting position acquisition delay	3/4	+ (13 month)	+ (12 months)	+ (18 months)	N/A	− (8 month)
	Walking acquisition delay	5/5	+ (4 years 4 month)	+ (4 years)	+ (4 years 4 month)	+ (2 years 6 month)	+ (4 years)
	Language delay	5/5	+	+	+	+	+
	Intellectual disability	4/4	N/A	+	+	+	+
	Macrocephaly	1/5	−	+	−	−	−
	Cerebellar symptoms	2/5	−	+	+	−	−

Behaviour	Behavioural abnormalities	4/5	+	+	+	+	−
	Auto or hetero-aggressivity	4/5	+	+	+	+	−

Sensory disability	Deafness	2/4	+	N/A	+	−	−
	Vision / ophtalmological abnormalities	3/5	+	+	−	+	−

Endocrine abnormalities	Precocious puberty	2/3	N/A	+	N/A	+	−

Dermatological features	Raynaud phenomenon	2/4	−	−	+	+	N/A
	Thin/ sparse hair	4/5	+	−	+	+	+
	Other dermatological features		+	+	+	+	−

Facial dysmorphic features	Facial dysmporphic features	5/5	+	+	+	+	+
	Plagiocephaly	2/5	Right side occipital plagiocephaly	−	−	Occipitoparietal plagiocephaly	−
	Forehead	3/5	Square forehead	−	Square forehead	Prominent forehead	−
	Eyes	5/5	Epicantus	Telecanthus	Synophrys, deep set eyes, upslanting palpebral fissures	Downslanting palpebral fissures, epicanthal folds	Deep set eyes
	Ears	5/5	Dysplastic ears	Posteriorly rotated ears	Prominent ears	Low-set and slightly prominent ears	Posteriorly rotated ears, dysplastic cupped ears
	Nose	3/5	Flat nasal root, hypoplastic alae nasi	−	Columnella below alae nasi	−	Broad nasal root
	Philtrum	2/5	−	Short, shallow philtrum	Long philtrum	−	−
	Mouth	3/5	−	Wide mouth, prominent lips	Broad mouth, red cheeks	Downturned corners of the mouth	−
	Tongue/teeth	3/5	Ankyloglossia	−	Diastema, carious teeth	Relative macroglossia	−

Abbreviations : N/A, non available

## Data Availability

This study did not generate code. The published article and supplementary material include all datasets analyzed during this study.
